# Donors With Previous Malignancy: When Is It Safe to Proceed With Organ Transplantation?

**DOI:** 10.3389/ti.2025.13716

**Published:** 2025-01-24

**Authors:** Vitor Turra, Joao Manzi, Sarah Rombach, Simone Zaragoza, Raphaella Ferreira, Giselle Guerra, Kendra Conzen, Trevor Nydam, Alan Livingstone, Rodrigo Vianna, Phillipe Abreu

**Affiliations:** ^1^ Miami Transplant Institute, Jackson Memorial Hospital, University of Miami, Miami, FL, United States; ^2^ HCA Healthcare–HealthOne Internal Medicine Residency Program, Sky Ridge Medical Center, Denver, CO, United States; ^3^ Department of Surgery, University of Colorado Anschutz Medical Campus, Aurora, CO, United States; ^4^ Sylvester Comprehensive Cancer Center, University of Miami, Miami, FL, United States

**Keywords:** risk, cancer, donor, malignancy, transplant surgery

## Abstract

The growing number of organ donors in the United States, from 14,011 in 2012 to 21,374 in 2022, highlights progress in addressing the critical issue of organ shortages. However, the demand remains high, with 17 patients dying daily while on the waiting list. As of August 2023, over 103,544 individuals are awaiting transplants, predominantly for kidneys (85.7%). To expand the donor pool, the inclusion of elderly donors, including those with a history of malignancies, is increasingly considered. In 2022, 7% of all donors were aged 65 and above, despite the complexities their medical histories may introduce, particularly the risk of donor-transmitted cancer (DTC). This review examines the challenges and potential benefits of using donors with known malignancy histories, balancing the risks of DTC against the urgency for transplants. A critical analysis is presented on current knowledge and the decision-making processes that consider cancer types, stages, and patient survival outcomes. The goal is to identify missed opportunities and improve strategies for safe and effective organ transplantation from this donor demographic.

## Introduction

Over the past few years there has been consistent growth in the number of organ donors with numbers rising from 14,011 donors in 2012 to 21,374 in 2022 in the United States [1, 2]. However, the problem of organ shortage remains a significant challenge with 17 patients on the waiting list losing their lives daily due to the unavailability of suitable organs [1, 2].

As of August 2023, the number of patients on the organ transplant waiting list reached 103,544 individuals [2]. Among the organ types, the kidney is the most prevalent, accounting for 85.7% of the patients, followed by those in need of a liver (9.8%), heart (3.2%), lung (0.9%), and other organs (0.4%) [2]. To address this critical need, there were 6,466 living donors and 14,903 deceased donors, totaling 21,369 individuals who donated organs. In 2022 alone, a total of 42,880 successful organ transplants were performed [1, 2].

As life expectancy continues to rise, a growing number of elderly patients appear as potential organ donors due to the necessity to increase the organ donor pool, even with marginal donors [3]. In 2022, 7% of all donors had 65+ years, the highest percentage ever [4]. However, this demographic often carries a history of comorbidities, including malignancies, which adds complexity to an already risk full procedure [5, 6]. One of the significant concerns is the possibility of transmitting diseases or malignancies from the donor to the recipient [7].

Donors with a history of cancer, the main focus of this review, are individuals who have been previously diagnosed and treated for malignancy, but whose cancer is considered cured or in remission at the time of organ donation. In contrast, donors with a known tumor prior to organ procurement or detected during procurement are individuals where the malignancy, such as renal cell carcinoma (RCC) or certain brain tumors, is actively identified either in pre-donation evaluations or during the retrieval process, raising immediate considerations for recipient safety and donor eligibility. Finally, donors with unknown or undetected tumors at the time of transplantation represent a distinct category, as these malignancies, such as malignant melanoma, are discovered only post-transplantation, often in the recipient, posing significant challenges in terms of retrospective diagnosis and management of transmitted cancer. These categories highlight the varying levels of risk and clinical decision-making required in the evaluation and use of organs from donors with malignancy-related considerations.

Even among donors with previous malignancy history, there are substantial differences in the risks [8–14]. Weighing the risks associated with donor-transmitted cancer (DTC) against the probability of a patient dying while waiting for a donation is a delicate and complex decision [15]. Clinical assessment, considering various factors such as the type and stage of cancer, as well as patient survival on the waiting list, is essential in determining the feasibility and safety of organ transplantation in such cases [8–14]. Even with optimal donor evaluation, there remains an inherent risk of tumor transmission, particularly as donor age increases, due to the higher likelihood of undetected or subclinical malignancies in older individuals.

This review will focus on known malignancy history to gather and present the most up-to-date knowledge about donor-transmitted cancer and critically analyze potential missed opportunities.

## Assessment of Transmission Risk

Reported rates of donor-derived cancer transmission to organ recipients vary significantly, ranging from 0 to 42 percent, depending on the data source [8, 9, 13, 16–18]. These high variations could be explained by older data relying on voluntary reporting of index cases and may, therefore, be prone to overestimation [11, 13, 14].

While the exact risk of transmitting any specific cancer from the donor to the recipient is often uncertain, it is possible to broadly assess the likelihood of transmission based on available knowledge regarding the cancer type, its stage, metastatic potential, and recurrence patterns in both transplant and non-transplant settings. Table 1 summarizes the main cancer types and stages and was prepared based on the most recent guidelines [8–14, 20].

**TABLE 1:** Risk assessment of major cancer types.

Risk classification categories	
	Minimal risk of transmission (<0.1%) – Likely to be acceptable for all organ types and recipients
	Low risk of transmission (0.1% to <2%) – Likely to be acceptable for many organ types and recipients
	High risk of transmission (≥10%) – May be acceptable in exceptional circumstances
	Unacceptable risk – Use of organs is not recommended in any circumstance

Breast cancer				
Ductal carcinoma *in situ*				
Stage Ia hormone-negative breast cancer, >5 years cancer free				
Stage Ib or higher hormone receptor-positive breast cancer				
Breast cancer diagnosed at retrieval				
Central nervous system Tumors (see Table 2 for more information)				
Primary brain tumors				
Secondary brain tumors Cerebral lymphoma				
Colorectal Carcinoma				
Carcinoma *in situ* of the colon or rectum				
Treated Stage I colorectal cancer (N0/M0), >5 years cancer free (except familial adenomatous polyposis)				
Stage I colorectal cancer diagnosed during retrieval Stage IIa colorectal cancer, >10 years cancer free				
Stage II or higher colorectal cancer with ≤10 years cancer free				
Renal Cell Carcinoma				
Renal cell carcinoma <1 cm, Fuhrman Grade I-II				
Renal cell carcinoma >1 and ≤4 cm, Fuhrman Grade I-II				
Renal cell carcinoma >4–7 cm, Fuhrman Grade I-II				
Renal cell carcinoma with extra-renal extension or Fuhrman Grade III-IV				
Lung Cancer				
*In situ* lung cancer				
Any history of metastatic lung cancer				
Prostate Adenocarcinoma				
Prostate cancer with Gleason score ≤6 or treated with Gleason score 7				
Recently diagnosed prostate cancer with Gleason score 7				
Prostate cancer with distant metastasis				
Skin Cancers				
*In situ* cutaneous melanoma *In situ* squamous cell carcinoma Basal cell carcinoma				
Cutaneous melanoma ≤0.8 mm (T1/N0/M0) completely resected or >0.8 mm (T2-T4/N0/M0) with >10 years cancer free				
Invasive cutaneous squamous cell carcinoma with nodal involvement or metastasis Cutaneous melanoma T2-T4 with ≤10 years cancer free with nodal involvement or metastasis Uveal or mucosal melanoma				
Thyroid Cancers				
Papillary thyroid microcarcinoma Differentiated thyroid tumors ≤4 cm limited to the thyroid (T1/T2)				
Newly diagnosed differentiated thyroid cancer >4 cm (T3, M0) or with extensive spread (T4), treated and ≥2 years cancer free				
Thyroid lymphomas, thyroid sarcomas, and other rare tumors of the thyroid Treated thyroid cancer with incomplete macroscopic tumor resection				
Others				
Choriocarcinoma				

Table 1 was built using the most recent guidelines around the world, including from the European Committee on Organ Transplantation, the Transplantation Society of Australia and New Zealand (TSANZ), the Advisory Committee on the Safety of Blood, Tissues, and Organs (SaBTO) of the UK Government. Guidelines from the USA, spain, and Italy were also included [9–15, 19].

## Primary Brain Tumors

Primary solid central nervous system (CNS) tumors may occasionally lead to death in circumstances where organ donation is possible [19]. Extracranial spread of brain tumors is rare, though there are reports of malignancy transmission to the recipients of organs from such donors [21–31].

Primary brain tumors are graded by the World Health Organization (WHO) from grade I to grade IV based on their biological behavior and prognosis [32]. Grade IV tumors are considered cytologically malignant and generally fatal, leading to the perception that they pose the highest risk of transmitting malignancy from donor to recipient [32]. However, several cases of organ transplants from donors with grade IV tumors have been reported without the transmission of malignancy to the recipients [19, 33].

For instance, a UK review of 448 recipients who received organs from 177 donors with primary CNS tumors, including 23 donors with grade IV gliomas and 9 with medulloblastoma, found no evidence of tumor transmission over a minimum follow-up period of 5 years [34]. Similarly, an Australian and New Zealand registry review of 46 donors (9 with high-grade tumors) who provided organs to 153 recipients did not identify any transmission events [35].

Another report from the United Network for Organ Sharing (UNOS) database, which included 642 recipients of organs from donors with CNS tumors, including 175 recipients from donors with high-grade tumors, documented a single case of disease transmission from a donor with glioblastoma multiforme to three recipients [25, 36]. Finally, in a Czech report of 42 donors (11 with high-grade tumors), no transmission was observed among 88 recipients monitored for 2–14 years [37].

**FIGURE 1:**
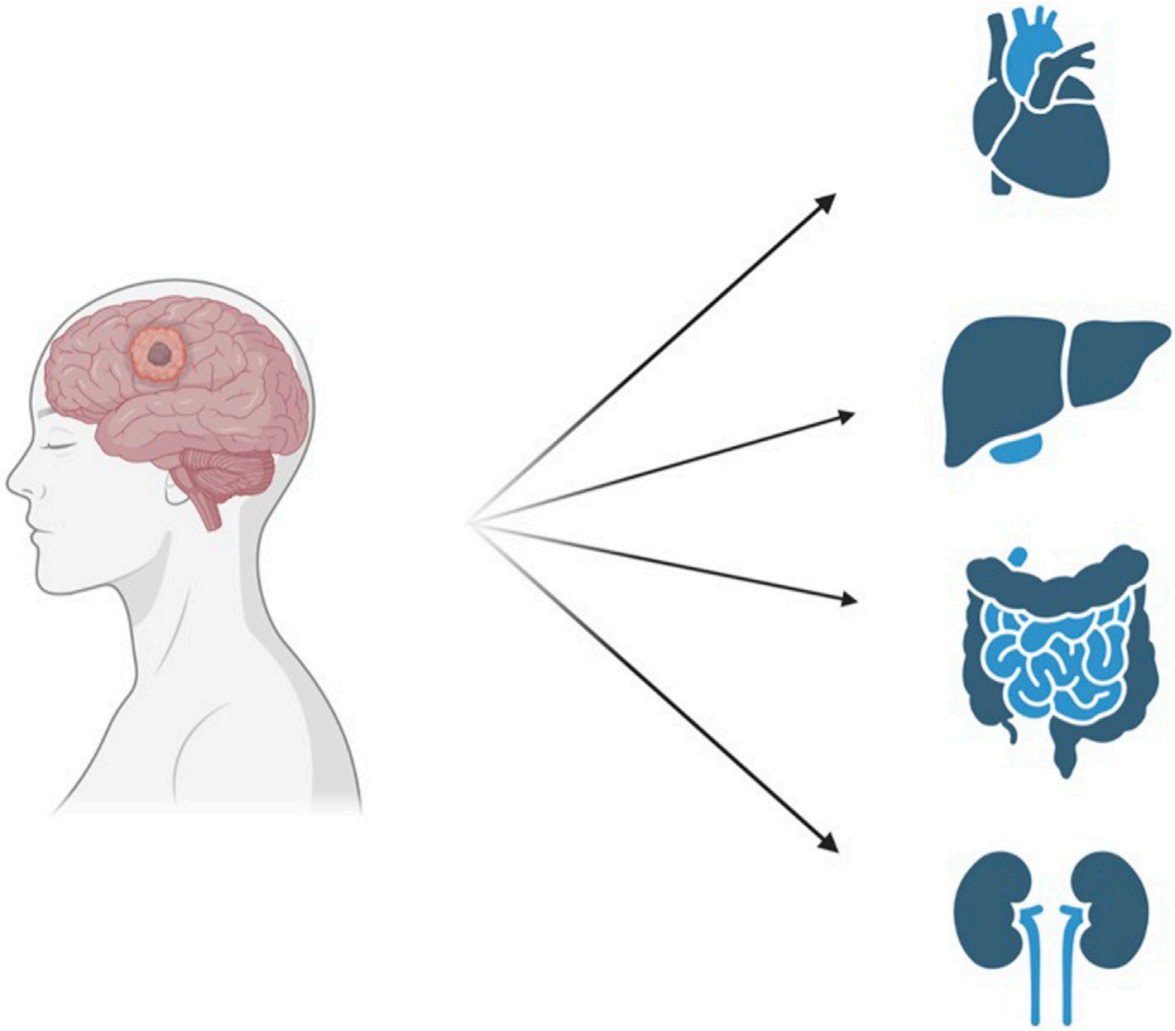
The still unexplored use of donors with previous malignancies can greatly contribute to an increase in the organ pool. Created with BioRender.com.

A more recent study also from the UK had a 10-year survival of transplants from donors with brain tumors of 65% (95% CI, 59%–71%) for single kidney transplants, 69% (95% CI, 60%–76%) for liver transplants, 73% (95% CI, 59%–83%) for heart transplants, and 46% (95% CI, 29%–61%) for lung transplants [19] which stays in proximity with UNOS national average without malignancy [38–41] (Figure 1). For example, kidney had a 78.15% (95%CI, 73.5%–82.7%) 10-year survival, liver had a 64.1%, heart had a 5-year survival of 80% and lung transplants had a 32.8% survival in 10 years [38–41].

Overall, The UK’s Advisory Committee on the Safety of Blood, Tissues, and Organs (SaBTO) [11] estimates the risk of tumor transmission from WHO grade I and II tumors to be minimal (<0.1%), the risk from grade III tumors to be low (0.1 to <2%) and the risk of transmission from grade IV tumors as 2.2% [10].

Subsequent reports, where WHO grading was adopted, have suggested that the risk of transmission is much lower [33–37, 42]. Of >77 donors with grade 4 CNS tumors donating to >338 recipients (>34 liver recipients), there was only one that transmitted cancer, with three recipients affected [25, 36]. Despite these favorable registry reports, there have been cases of CNS tumor transmission, including 6 in LT recipients [22, 23, 25, 27, 29, 31, 43–45].

In addition to the reported risks, other factors show clinical significance pertaining to CNS malignancy transmission. Interventions such as brain irradiation, chemotherapy, previous craniotomy, and ventriculoperitoneal shunt procedures may increase the risk of transmitting CNS malignancy from donors to recipients [11, 43, 46]. These interventions potentially breach the blood-brain barrier, facilitating tumor spread. However, it is challenging to differentiate between causality and coincidence. It is possible that certain interventions are more commonly employed in tumors that are more prone to spreading.

One important factor is that the presence of brain metastases can sometimes be incorrectly diagnosed as primary CNS tumors or intracranial hemorrhage, and organ transplantation from these donors has been associated with a poor prognosis for the recipients [46]. A study involving 42 recipients of organs from patients with misdiagnosed primary CNS tumors revealed that 74% of the recipients developed a malignancy derived from the donor, and 64% developed metastatic disease [46]. The 5-year survival rate for these recipients was only 32% [46]. Therefore, in cases where donors present with unexplained intracranial hemorrhage or suspected primary CNS neoplasm without a biopsy, it is crucial to consider conducting an evaluation specifically for metastatic disease [46].

The risk assessment of CNS cancers, specifically pertaining to organ transplantation shown in Table 2 has been conducted by utilizing the recommendations provided by the Advisory Committee on the Safety of Blood, Tissues, and Organs (SaBTO) [10] and the UNOS recommendations [11]. These assessments have incorporated findings from the SaBTO report, and the outcomes of more recent studies conducted, including those within the United Kingdom [19, 34, 47]. This led to a revised understanding of the risks associated with CNS tumors, but still deficient in quantity and quality of evidence.

**TABLE 2:** Risk assessment of CNS tumors.

Absolute contraindications
• Primary cerebral lymphoma • All secondary intracranial tumors • Any cancer with metastatic spread
Intracranial tumors with Intermediate risk of cancer transmission (2.2%) include WHO Grade 4 tumors
• Atypical teratoid/rhabdoid tumor • Choriocarcinoma • Diffuse midline glioma, H3K27 M-mutant • Embryonal tumor (all subtypes) • Giant cell glioblastoma (old classification) • Glioblastoma (IDH wild type and IDH mutant) • Gliosarcoma • Malignant peripheral nerve sheath tumor (MPNST) – grade 4 • Medulloblastoma • Medulloepithelioma • Pineoblastoma
Intracranial tumors with a lower risk of cancer transmission (<2%) include WHO Grade 3 tumors
• Anaplastic CNS tumors • Choroid plexus carcinoma • Ependymoma: RELA fusion-positive • Haemangiopericytoma/solitary fibrous tumor • Papillary tumor of the pineal region • Pineal parenchymal tumor of intermediate differentiation • Malignant peripheral sheath tumor grade 3
Intracranial tumors with minimal risk of cancer transmission (<0.1%)
• Low-grade CNS tumor (WHO grade I or II) • Primary CNS mature teratoma

The risk assessment of CNS cancers, specifically pertaining to organ transplantation shown in Table 2 has been conducted by utilizing the recommendations provided by the Advisory Committee on the Safety of Blood, Tissues, and Organs (SaBTO) [10] and the UNOS recommendations [11]. These assessments have incorporated findings from the SaBTO report, and the outcomes of more recent studies conducted [19, 34, 47].

## Breast Cancer

Breast cancer is the most frequent cancer in females and is associated with the highest mortality [48]. Organs from donors with a history of invasive breast cancer should only be considered when a low risk of transmission criteria is observed because of the potential for metastasis and late recurrence [49, 50]. A history of Stage I, T1A, node-negative, hormone receptor-negative breast cancer may still be viable in a donor that has had full treatment and complete remission with follow-up >5 years [51]. Any other type of invasive breast cancer is considered a high risk (>10%) of malignancy transmission, regardless of the disease-free interval [13].

Hormone-positive breast cancer poses a high cumulative risk of recurrence at 20 years post-treatment [49, 50]. Given this fact, donors with this type of cancer have a high transmission risk [9]. Lobular breast cancer and ductal carcinoma provide a similar risk of recurrence [52], so it is possible to group them together under Stage I breast cancer with >5 years of recurrence-free survival for risk assessment. In the event of a known history of invasive breast cancer but insufficient data, either pathologic or clinical, donation should only be considered for recipients facing an imminent threat to life [9]. Invasive breast cancer diagnosed during retrieval poses an unacceptable risk to potential transplant recipients [13].

## Renal Carcinoma

Literature shows documentation of successful kidney transplantation after renal cell carcinoma resection for tumors <4 cm detected at organ retrieval [53–55]. In one study following 21 kidneys with tumors from 0.1 to 2.1 cm, as well as 47 contralateral kidneys and 198 non-renal organs, no cases of malignancy transmission were identified [53]. Another study showed no cases of transmission in 97 kidney transplantations after RCC resection <4 cm, although there was one case in the transplant of 22 contralateral kidneys [54]. In the case of well-differentiated RCC, the risk of transmission was assessed as minimal (<0.1%) in tumors ≤1.0 cm in size, or low (<2%) for tumors >1.0 cm to ≤4 cm in size [10]. Therefore, all organs are considered for transplantation, including the affected kidney, after resection on as RCC <4 cm with Fuhrman grade I-II, when satisfactory margins are achieved [10, 11, 56]. Outside of organ retrieval, if RCC diagnosis was less than 5 years before organ donation, the same risks for RCC diagnosed during organ retrieval apply. For patients with RCC >5 years with appropriate follow-up, theoretical risks may be even lower [9]. Donors with RCC 4–7 cm with Fuhrman I-II, with higher than 5 years cancer-free interval may be considered for non-renal organs [57] Any history of invasive RCC or Fuhrman grade III-IV represents an unacceptable risk [13].

## Primary Liver Tumors

Liver, biliary, or pancreatic cancers that are diagnosed during organ retrieval provide an unacceptable risk of malignancy transmission in organ transplantation [9]. Even if identified in treated history, they are usually also considered unacceptable risks given the aggressive nature and high recurrence of these cancers [9].

However, benign liver tumors are relatively common, occurring in up to 20% of the general population [58] the most frequent lesions being hepatic hemangioma (HH), focal nodular hyperplasia (FNH), and hepatocellular adenoma (HCA and are safe to transplant, so it’s essential to differentiate between a tumor is malignant before ruling out donation [58, 59]. Some studies exist showing cancer transmission in liver, biliary, or pancreatic cancer [42, 60–63].

## Malignant Melanoma

Melanoma of the skin represents 5% of all new cancer cases in the US [64]. Melanoma is known for its potential transmission from donor to recipient during transplantation [36, 65–68] particularly pronounced when the diagnosis is overlooked in the donor, leading to significant implications [36, 57, 69–73]. The prevalence of melanoma as a tumor type is high, marked by early micro metastasis and the inherent challenge of detection [74, 75]. Invasive melanoma constitutes around 30% of reported cases of donor-related cancers [18, 76] with fatal consequences, as it correlates with a high recipient mortality rate, estimated at approximately 60% [7]. The level of risk associated with the transmission of cutaneous melanoma hinges on factors like Breslow thickness and the stage of melanoma at the time of diagnosis and treatment [77]. Notably, *in situ* cutaneous melanoma, being non-invasive, presents minimal chances of donor-derived transmission due to the absence of metastatic risk associated [65, 78, 79].

Invasive cutaneous melanoma is considered a high to unacceptable risk of transmission as it may recur regardless of many years of disease-free interval and poses a theoretically higher threat on immunosuppressed patients, given that on non-immunosuppressed individuals, the lifetime risk of recurrence is greater than 2% for T1a (<0.8 mm thickness) and greater than 10% in T1b (0.9–1.0 mm) [80–82]. Another hazard of melanoma is its spread to distant sites, even during the early stages of the disease, with cells that may stay dormant and undetectable for many years after primary resection [83]. If transplanted, these cells may lead to metastatic growth in an immunosuppressed patient [66, 84–86], with high mortality rates [87, 88]. Similarly, uveal and mucosal melanoma pose an unacceptable risk to donation, given a high risk of undetected micro metastases, regardless of the length of disease-free survival, as does cutaneous melanoma with a history of nodal involvement or distant metastases [89–91].

Considering all these factors, there are instances where organs from donors with melanoma, other than *in situ*, may be used under exceptional life-or-death circumstances [13]. As always, this decision must be based on a thorough assessment of risk status, with ample information available, and always accompanied by the informed consent of the recipient.

## Prostate Cancer

Prostate cancer provides a minimal-to-low risk of malignancy transmission given, as with many other types of cancer, its confinement to the original organ [92]. It is one of the most prevalent cancers accounting for 14.7% of all new cancer cases in the U.S. In 2023, there were an estimated 4,956,901 men living with prostate cancer in the World [48, 93]. A study conducted on organ donors showed that 23% of those aged 50–59 years, 35% of those aged 60–69, and 46% of those aged 70–81 years had undiagnosed prostate cancer [94]. However, there was no evidence of higher prevalence of prostate cancer among transplant recipients relative to the general male population [95, 96].

The Gleason score is a valuable tool when deciding to proceed with the donation [97, 98]. A Gleason score of 6 provides an almost-zero risk of transmission [99]. A donor with a history of a Gleason 7 prostate cancer may also be considered minimal risk, provided the tumor was organ-confined and the donor has been cancer-free for more than 3 years [92, 100]. Analyzing 120 reports of transplants coming from donors with confirmed prostate cancer, only one case was identified [101], and that came from a donor later found to have metastatic disease [102]. A meta-analysis concluded that the risk of remaining on the waiting list was higher than the risk of transmission in transplants with a donor with prostate cancer [92].

## Primary Lung Carcinoma

There are registry and case reports of occult donor transmission with kidney transplantation, highly fatal outcomes, and very aggressive behavior from donor-transmitted lung cancer [57, 103–105]. Benign pulmonary nodules – such as hamartomas and papillomas – are relatively common, especially after 45 years of age and account for more than 95% of all pulmonary nodules [106]; hence it is important to distinguish between benign tumors in the lung and lung cancer in the donor.

Transmission of lung cancer to liver transplant recipients has been reported with fatal consequences in 2 cases, including 1 undergoing urgent transplantation when the adenocarcinoma was found on donor autopsy [107, 108]. There are also reports of transmission in several registry studies [17, 18, 20, 60, 69, 109]. In contrast, there are a few reports of donor lung cancer not being transmitted to liver transplant recipients [17, 20, 110]. Still, lung cancer at any stage (excluding *in situ* – high risk) [13] is considered an unacceptable risk [9, 13].

## Colorectal Cancer

Colorectal cancer is common in the population and a common cause of mortality [48]. The liver is the most frequent site of metastasis [111]. A 2003 US consensus agreed on the use of Stage I – T1, node-negative – colorectal cancer individuals as organ donors given the low risk of nodal or metastatic disease associated [112]. For individuals with Stage I familial adenomatous polyposis who are potential donors, caution should be exercised when considering pancreas transplantation due to an elevated risk of duodenal cancers [13]. However, under specific circumstances and clinical assessment, transplantation of certain other organs might still be viable [9]. In cases where Stage II or higher colorectal cancer is detected either during retrieval or in the donor’s medical history, with a cancer-free period of up to 10 years, the potential for transmitting cancer to recipients is deemed unacceptable [11, 13].

However some more recent studies suggest the risk might be lower [110, 113]. As new forms of cancer targeting appear [114] and recently showed that patients with this type of cancer may have a good prognosis [115, 116] with effective surveillance [117], new data on colorectal cancer and its safety should appear in the following years. It is important that pathology reports are made available to accurately determine the stage of cancer before proceeding with transplantation. During retrieval procedures, surgeons should meticulously inspect all intra-abdominal and intra-thoracic structures for any suspicious lesions [15, 109].

## Thyroid Tumors

Approximately 90% of thyroid cancers are either papillary (80%) or follicular (10%) [118], usually with only localized spread [48]. The relative survival rate is 97% in a 5-year interval, if not in advanced stages [48, 119]. Distant metastases develop in 5%–23% of differentiated thyroid cancers, typically in the lungs and bones [120]. Post-operative risk of recurrence after tumor resection is low when the tumor does not have aggressive histology [121].

The risk of malignancy transmission in differentiated thyroid cancer varies depending on the size of the tumor and the spread, with cancers up to 4 cm providing a minimal risk of transmission if confined to the thyroid, even if only detected at organ retrieval [9, 11, 13]. On an important note, thyroid cancers are not affected by immunosuppression, which means that pre-existing thyroid cancers do not show increased rates of progression, and the incidence of thyroid cancer is not elevated in recipients [96, 122]. Additionally, even in the event of donor-derived transmission, metastatic differentiated thyroid cancer can still be treated with curative therapy, depending on histology [120].

## Other Cancers

While the main cancers associated with organ donation are well-documented, consideration should also be given to other less commonly reported malignancies, such as ovarian, cervical, and pancreatic cancers. These malignancies pose unique challenges due to their aggressive nature and potential for microscopic metastases. However, the current literature lacks sufficient data to assess the transmission risks or to establish evidence-based recommendations for the utilization of organs from donors with these types of cancers. As such, further studies and case reports are needed to better understand the risks and outcomes associated with these malignancies in the context of organ transplantation.

## Discussion

History of malignancy or, in some cases, an active malignant disease in the potential donor should not automatically be a veto to organ donation. The estimated risk of tumor transmission should be balanced against the benefit of the transplant for recipients. Donor-transmitted cancer is still an area to be better understood, with few quality studies showing varied results that could either underestimate or overestimate the probability of developing DTC [123–125].

As the donor waiting lists continue to rise and general life expectancy tends to get older, reevaluating the risk of DTC could provide a powerful ally in increasing the donor pool, with many more donors becoming available. Currently, there is still a feeling of missed opportunities in perceived high-risk transplants that retrospectively have not shown the same risk, especially regarding CNS tumors [126], that’s have historically been put in a >10% risk category [11] and are now reduced to a 2.2% risk [8–10, 13]. There is expectation concerning what other risk reductions are viable, but more detailed data, including reliable reporting of transmission events, is necessary to include, as of now, high-risk malignancies in a potential donor list and to allow a more evidence-based decision process.

The frequently urgent nature of organ transplantation often precludes the possibility of obtaining all of the desired information, and the physician must weigh available clinical data and published experience along with the medical condition and desires of the patient in arriving at the best possible decision. Although a certain transmission risk will remain in many cases, selected patients will benefit from these organs more than if they stayed on the waiting list.

It is important to notice, however, that even without a prior history of neoplasm, there is still a chance of donor-origin cancer (DOC) of 0.06%, as it was concluded by a study with 30,765 transplants conducted in the UK [17]. In this study of the 18 recipients who developed DOC from 16 donors (0.06%): 3 were DDC (donor-derived cancer - posterior growth of malignancy after transplantation, derived from donor cells), and 15 were DTC (donor-transmitted cancer). Of the 15 DTCs, 6 were renal cell cancer; 5 lung cancer; 2 lymphoma; 1 neuroendocrine cancer; and 1 colon cancer. This represented an unavoidable, but low risk of DOC in every transplant made [17], which should be taken into consideration when weighing the risks.

Noticeably, no guidelines exist on retransplantation in DTC events. Decisions should be made on a case-by-case basis with a multidisciplinary approach and after discussion with the patient or relatives. Retransplantation may be reasonably considered when the tumor identified in the donor is deemed of intermediate or high risk of transmission.

## Conclusion

An individualized clinical judgment for using organs from donors with malignancy should be made and presented to the recipients, including the risk of not proceeding with transplantation, with fully informed consent being mandatory where the risks are higher than standard expectations.

This review stands to bring back the focus on DTC, urging for a more extensive evidence base providing more accurate and clinically relevant recommendations to aid the patient’s physician in a more secure clinical decision, as well as providing an answer to an ever-higher donor waiting list.
